# A strategy to balance location privacy and positioning accuracy

**DOI:** 10.1371/journal.pone.0304446

**Published:** 2024-05-30

**Authors:** Li He, Junqing Liu, Peiyao Du

**Affiliations:** School of Computer Science and Technology, Chongqing University of Posts and Telecommunication, Chongqing, China; National University of Sciences and Technology NUST, PAKISTAN

## Abstract

In privacy protection methods based on location services, constructing anonymous areas using location information shared by collaborative users is the main method. However, this collaborative process not only increases the risk of mobile users’ location privacy being leaked, but also reduces positioning accuracy. In response to this problem, we propose a balancing strategy, which transforms the problem of protecting mobile users’ location privacy and improving positioning accuracy into a balance issue between location privacy and positioning accuracy. The cooperation of mobile users with different collaborating users is then modeled as an objective optimization problem, and location privacy and positioning accuracy are evaluated separately to make different selection strategies. Finally, an optimization function is constructed to select the optimal selection strategies. Experimental results show that our proposed strategy can effectively achieve the balance between location privacy and positioning accuracy.

## Introduction

With the rapid development of the Internet of Things and 5G/6G networks, smart mobile devices have accelerated their upgrading [1, 2]. The popularity of smart mobile devices has also promoted the application of location-based services (LBS) [3]. LBS pertains to the utilization of geographic location coordinates and associated data derived from mobile devices, with the objective of furnishing users possessing such devices with information resources and foundational services pertinent to their specific locations. The scope of LBS applications encompasses cartographic utilities, points of interest retrieval, and navigation, among others. Early LBS systems were mainly used in military and civilian fields involving important national interests. At present, LBS has been widely used in situation awareness, traffic navigation, business services, leisure and entertainment, and other fields [4]. Users can obtain points of interest (POI) near the location, such as the nearest restaurants, supermarkets, hospitals, etc., by sending LBS service requests. It can be seen that location-based services have penetrated into all aspects of people’s daily lives, and thoughtful and meticulous services make people increasingly inseparable from LBS.

However, in order to obtain necessary information services, users need to send their location information to the LBS server, and the server returns corresponding query results to the user based on the uploaded location information. At the same time, because the LBS server has the characteristics of honest but curious [5], it can make inferences based on the private information data uploaded by users, which may cause security issues for users [6]. For example, a malicious attacker may use private data to analyze the locations where users often stay, and determine whether the locations where users often stay during a specific period of time are home addresses and work locations. If malware cannot be prevented from spreading on the LBS server, or the LBS server does not have intrusion detection methods to detect malicious traffic, then this information will be cracked by malicious attackers, and users will face serious security and property threats [7–9].

In order to protect user location privacy, users need to communicate and cooperate with surrounding cooperating users to send the location information of multiple users to the server. However, working with more users means a reduction in positioning accuracy, thus affecting the accuracy of user location-based services. Therefore, it is crucial to balance location privacy and positioning accuracy for mobile users.

**Fig 1:**
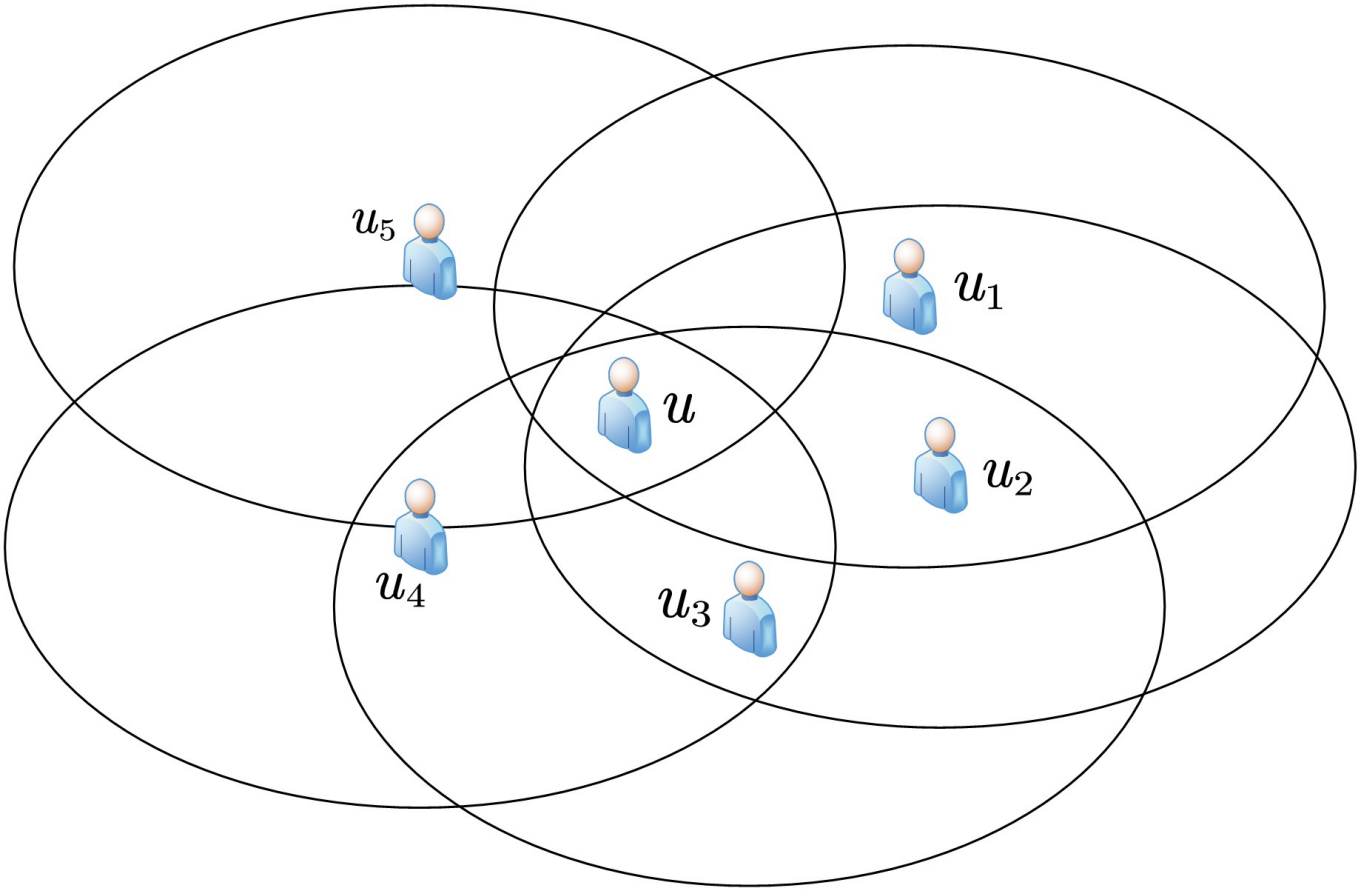
Example of requesting user cooperation.

**Example** Taking Fig 1 as an example, if the mobile user *u* cooperates with *u*_1_, *u*_2_, *u*_3_, *u*_4_, and *u*_5_ simultaneously, it can be determined that *u* is within the area jointly covered by the *u*_1_, *u*_2_, *u*_3_, *u*_4_, and *u*_5_ response areas. This area is referred to as the inferred area in this paper, which is used to measure the accuracy of the inferred positioning accuracy. The distance between the cooperative user and the mobile user indicates whether the actual positioning is accurate, that is, the average distance between cooperative users and the mobile user is used to measure the accuracy of actual positioning accuracy. The accuracy of user positioning is determined by both the inferred positioning accuracy and the actual positioning accuracy. The degree of location privacy protection for the mobile user is also the same, and cannot be simply measured by Yes or No. The convex hull area formed by *u*_1_, *u*_2_, *u*_3_, *u*_4_, and *u*_5_ in Fig 1 is referred to as privacy area in this paper. The risk of the mobile user location information leakage depends on the size of the privacy area, because the smaller the privacy area, the closer the positions between cooperative users, thereby increasing the probability of mobile user location being discovered by attackers, and the higher the risk of location information leakage. In this paper, the key to protecting user privacy is to maximize the privacy area, as the larger the privacy area, the less likely the mobile user’s location will be discovered by attackers. Therefore, cooperative users should stay as far away as possible from the mobile user. But this will reduce the actual positioning accuracy of user and improve their inferred positioning accuracy. In addition, the shorter the average distance between cooperative users and the mobile user, the higher the actual positioning accuracy of the mobile user. However, this increases the risk of user location privacy leakage. Reducing the actual positioning accuracy in turn leads to an increase in inferred positioning accuracy, which not only lowers the user’s positioning accuracy but also increases the risk of location privacy leakage. Therefore, it is necessary to improve the actual positioning accuracy of users and reduce their inferred positioning accuracy while ensuring the privacy and security of user location. Therefore, we regard this issue as a balance among location privacy, actual positioning accuracy, and inferred positioning accuracy.

In response to the above problems, we propose a strategy to balance location privacy and positioning accuracy. The contributions of this paper are summarized as follows:

- We proposed a model to balance location privacy and positioning accuracy(BLPPA), and proved that it is NP-hard.
- We design a privacy evaluation model and a positioning accuracy evaluation model. It is proposed to use privacy areas to evaluate location privacy, while using actual distance and inferred areas to evaluate actual positioning accuracy and inferred positioning accuracy respectively. An optimization function is established to select the optimal decision to achieve a balance between location privacy and positioning accuracy.
- Based on the public real data set, experimental comparisons were conducted with existing solutions to verify the effectiveness of our designed model in balancing location privacy and positioning accuracy.

The remainder of this paper is organized as follows. Related work section introduces related work in the field of location privacy, and Problem statement section analyzes and formulates the BLPPA problem, proving the difficulty of the problem. Privacy and positioning accuracy assessment section evaluates location privacy and positioning accuracy respectively, and proposes an optimization function to find the optimal strategy. Experimental results section conducts experimental analysis. Conclusion section summarizes the full text.

## Related work

In order to solve the problem of location privacy leakage, researchers have proposed many algorithms, which are summarized as follows.

In the research on location privacy protection methods, anonymous methods are the more mature and most commonly used technology. Among the anonymous methods, *K*-anonymity [10] is one of the most classic algorithms.

*K*-anonymity is a data desensitization method. The core idea is to generalize quasi-identifiers so that any piece of data cannot be distinguished from at least *K* − 1 other pieces of data. Gruteser et al. [11] first introduced the *K*-anonymity concept of relational databases into the field of LBS privacy protection and proposed location *K*-anonymity. It means that the position in the LBS query corresponds to an area containing at least *K* different users, and the attacker cannot distinguish the real query user from these *K* users. A larger *K* value indicates a higher degree of privacy protection, but the query accuracy will be lower, resulting in lower quality of service (QoS).

In recent years, researchers have been continuously proposing improvements and optimizations to *K*-anonymity techniques. Xing et al. [12] proposed a location privacy protection method based on double *K*-anonymity, which introduces a cloud server as a trusted third-party to isolate the direct communication between the user and the service provider, and at the same time reduces the relevance of the identity to the request through the method of substitution and combination, in order to hide the user’s location and request information. Peng et al. [13] proposed a multidimensional privacy protection scheme that provides comprehensive protection for user privacy without the need for a trusted third party. The scheme employs a semi-trusted intermediate entity to perform user anonymization and blind filtering of results, utilizes Hilbert curves to transform the user’s location, and uses encryption to preserve the user’s query.

However, the above methods all rely on a third-party central server, which may store the user’s real location information, and all queries submitted must go through it, which itself may become a bottleneck for system service performance and failure. In order to solve the shortcomings of centralized architecture, researchers have proposed distributed architecture [14–16]. Cui et al. [14] proposed a novel architecture that builds a non-localized LBS based on a distributed architecture, allowing mobile users to access the LBS without revealing their location. Furthermore, a technique to evaluate mobile user privacy and utility is proposed to achieve a balance between them. Shi et al. [15] considered that cooperative nodes would incur privacy costs when reporting their location information, and proposed a feasible incentive mechanism based on contract theory to reward cooperative nodes and ensure the expected positioning accuracy of the target node. Zhang et al. [16] proposed a cache-based double *K*-anonymity location privacy protection scheme, which reduces the load on user devices by applying multi-level caching and protects location privacy through double anonymity.

In order to address the issue of untrustworthy collaborators in distributed architectures, researchers have proposed conducting trust assessments on cooperative users to identify malicious users who disguise themselves as normal users and may engage in malicious operations [5, 17–20]. Luo et al. [17] and Li et al. [5, 18] proposed a blockchain based trust location privacy protection scheme in VANET. This scheme designs a trust management method based on Dirichlet distribution by analyzing the different requirements of requesting and cooperative vehicles in the process of constructing anonymous camouflage areas, so that requesting and cooperative vehicles only cooperate with the vehicles they trust. Liu et al. [19] proposed a blockchain-based TM scheme together with a conditional privacy-preserving announcement protocol (named as BTCPS). By the use of group signatures in anonymous aggregate vehicular announcement protocol, the reliability of announcements can be maintained without revealing users’ privacy in the non-fully-trusted environment. Feng et al. [20] proposed a trusted CAC scheme called TCAC to protect the location privacy of vehicles. With the trust mechanism, multiple anonymizers in adjacent vehicular regions can be selected to construct the cloaking area in a cross-region manner. Min et al. [21] proposed a location privacy protection method in 3D space based on geo-indistinguishability, which develop a mechanism of three-variates Laplacian to generate perturbed locations considering the locations’ X, Y, and Z-coordinates simultaneously, guaranteeing geo-indistinguishability. Furthermore, the truncation of the Laplace mechanism was further studied to limit the generated perturbation locations to specific regions. Kim et al. [22] used the perturbation mechanism of Geo-I to obfuscate user location information, and then proposed an expectation-maximization (EM) algorithm and the deep learning based approaches to accurately calculate the density distribution of LBS users while preserving the privacy of location datasets.

In the field of location privacy protection, differential privacy [23] is also a popular research direction, which is characterized by being unaffected by attackers with background knowledge. Andrés et al. [24] proposed Geo-indistinguishability, where noise is added to the user’s true location so that the final published user’s location is within a circular range of the true location, making it impossible for the service provider to get the user’s true geographic location from the collected location information. Li et al. [25] proposed an enhanced privacy definition beyond Geo-indistinguishability, combining with differential private indexing mechanism to design a new mechanism to realize this definition, by guaranteeing that the user’s pseudo-location is reasonable to prevent the user’s location perturbation behavior from being recognized. Wang et al. [26] proposed a location privacy preserving algorithm with location clustering and differential privacy, which firstly divides the continuous locations into different clusters, and then adds Laplacian noise to the stationary points and centers of mass within the clusters to protect the user’s location privacy.

In the field of industrial Internet of Things, some research on privacy protection can also bring some inspiration. Wu et al. [27] proposed a privacy-preserving offloading scheme based on stochastic game theory considering multiple access points. In terms of privacy, the privacy risks caused by the offloading preferences of different edge nodes are studied, and the privacy entropy is used to evaluate the privacy protection level. Shen et al. [28] proposed a privacy protection model based on signaling game. This paper first derives the optimal privacy protection strategy of the model from a theoretical perspective. And a signaling Q-learning algorithm is designed to formulate the optimal privacy protection strategy by combining the Bayesian rule and the Q-learning approach. Wu et al. [29] first define the cumulative privacy amount for each IIoT user and trigger the privacy protection mechanism when the cumulative privacy amount exceeds the set privacy threshold. The offloading data generated by the IIoT user is then transferred to local processing, and finally, the cumulative privacy amount of the IIoT user is reduced.

The comparison of location privacy protection algorithms is shown in Table 1. Through the analysis of current research results, it can be found that the sharing of location information increases the risk of location privacy leakage for mobile users. At the same time, it also reduces the positioning accuracy. Therefore, we conduct research on this issue and propose solutions.

**Table 1:** Comparison of location privacy protection algorithms.

References	Methods	Degree of location privacy protection	Accurate level of positioning accuracy
[12]	double k-anonymity	medium; no guarantee that cloud servers are completely trustworthy	not considered
[13]	encryption	medium; semi-trusted middle entity risk privacy leakage	not considered
[14]	a novel architecture	high; depend on the number of servers accessed simultaneously	medium; depending on privacy zone size
[15]	differential privacy	high; depend on how much noise is added	medium; depending on the degree of incentive of the incentive mechanism
[16]	double k-anonymity	high; depend on cache size	not considered
[5, 17–20]	blockchain	high; depend on the trust value threshold	not considered
[21–26]	differential privacy	high; depend on cache size	not considered
ours	k-anonymity	high; depend on the size of the privacy area, it can balance positioning accuracy while also protecting location privacy to a high degree.	high; using actual positioning accuracy and inferred positioning accuracy to comprehensively evaluate the positioning accuracy of mobile users

## Problem statement

### Preliminary

#### Convex hull [30]

The minimum convex polygon encompassing all the points in a given point set Q is referred to as the convex hull. Since the convex hull problem investigates how to construct the smallest convex polygon that can enclose the given point set, we employ convex hull computation to determine the privacy area for cooperative users.

#### 3-SAT problem [31]

The 3-SAT problem is a Boolean satisfiability problem. Given a Boolean expression, it is necessary to find a variable assignment that makes the expression result true. If there exist assignments of variables as true or false that result in the Boolean expression evaluating to true, then the expression is satisfiable. If no such assignments exist, and for all possible variable assignments, the expression always evaluates to false, then the expression is unsatisfiable.

#### Haversine formula [32]

The Haversine formula is a method for calculating the distance between two points on a great circle on the Earth’s surface based on their longitudes and latitudes. It approximates the Earth as a sphere with a radius of R. This formula allows for the calculation of the great circle distance between any two points, A and B, on the Earth.

#### Heron’s formula [33]

The Heron’s formula is a mathematical formula used to directly calculate the area of a triangle based on the lengths of its three sides.

### Problem analysis

**Fig 2:**
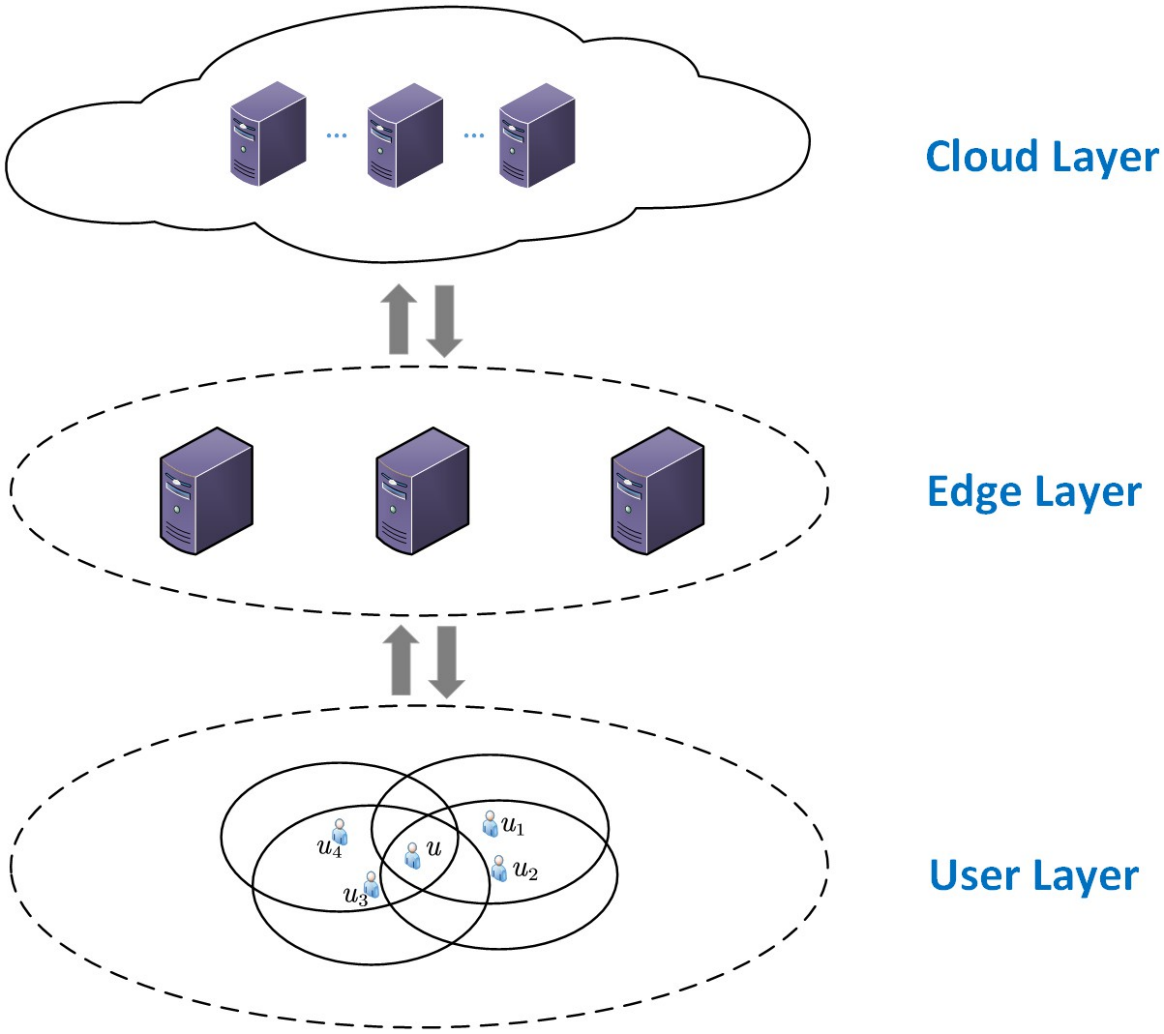
System architecture.

When a mobile user has multiple cooperating users to cooperate with, a selection strategy needs to be made to determine which cooperating users the mobile user cooperates with to build an anonymous area. Taking the mobile user *u* in the user layer in Fig 2 as an example, there maybe be sixteen different selection strategies, namely {{⊘}, {*u*_1_}, …, {*u*_3_}, {*u*_1_, *u*_2_}, …, {*u*_2_, *u*_3_}, {*u*_1_, *u*_2_, *u*_3_}, …, {*u*_1_, *u*_2_, *u*_3_, *u*_4_}}. These selection strategies result in different positioning accuracy and privacy area. For example, the selection strategy {*u*_1_, *u*_2_, *u*_3_, *u*_4_} indicates that *u* cooperates with all surrounding cooperative users at the same time, thus producing the largest privacy area. However, this selection strategy produces the highest inferred positioning accuracy. Too high inferred positioning accuracy will lead to too small inferred area and attacks. Combined with surrounding buildings and other information, the real location of the mobile user can be easily inferred, and this selection strategy produces the lowest actual positioning accuracy. In order to reduce the risk of the location being inferred and to improve the actual localization accuracy, *u* will work with as few and as close collaborating users as possible to increase the inferred area size and shorten the average distance, but this leads to a reduction of the privacy area, making the anonymous area construction less effective and not conducive to privacy protection. Therefore, the solution to the BLPPA problem must achieve a balance among privacy area, actual positioning accuracy, inferred positioning accuracy generated for *u*. The main symbols and their interpretations in this paper are shown in Table 2.

**Table 2:** Key notations.

Notation	Description
*u*	the mobile user *u*
*m*	number of cooperative users
*N*(*u*) = {*u*_1_, …, *u*_*i*_, …, *u*_*m*_}	set of *u*′ cooperative users
*u*_*i*_ ∈ *N*(*u*)	cooperative user *u*_*i*_
*a* = (*u*_1_, …, *u*_*i*_, …, *u*_*k*_)	selection strategy *a*
***a*** = {*a*_1_, …, *a*_*i*_, …, *a*_2^*m*^_}	BLPPA strategy ***a***
*ad*(*a*)	actual distance produced by *a*
*ia*(*a*)	inferred area produced by *a*
*pa*(*a*)	privacy area produced by *a*
λ_*ad*_	weight of actual distance
λ_*sa*_	weight of inferred area
λ_*pa*_	weight of privacy area
*p*_*i*_	point *p*_*i*_
*circles*(*u*_*j*_)	coverage of *u*_*j*_
*d*(*u*_*i*_, *u*_*j*_) or *d*(*p*_*i*_, *p*_*j*_)	distance between *u*_*i*_ or *p*_*i*_ and *u*_*j*_ or *p*_*j*_
*r*	coverage radius of *u*
*R*	earth radius
*S*(Δ*p*_*i*_*p*_*j*_*p*_*k*_)	triangle area formed by points *p*_*i*_,*p*_*j*_ and *p*_*k*_
$\overset{⌢}{S}(p_{i}O_{p_{i}p_{j}}p_{j})$	sector area formed by points *p*_*i*_,*p*_*j*_ and $O_{p_{i}p_{j}}$
$\overset{⌢}{S}(p_{i}p_{j})$	arched area formed by points *p*_*i*_,*p*_*j*_

### Problem formlation

**Definition 1 (Selection Strategy).** Given the mobile user *u* and the set of cooperative users *u*_*i*_ ∈ *N*(*u*), a selection strategy represents the collaboration of the mobile user *u* with any cooperative user *u*_*i*_. If *u* collaborates with *k* cooperative users, the selection strategy can be denoted as *a* = (*u*_1_, …, *u*_*i*_, …, *u*_*k*_), *i* = 1, 2, …, *k*;*k* ≤ *m*.

**Definition 2 (BLPPA Strategy).** The *u*’s BLPPA strategy is composed of a set of selection strategies represented by **a** = {*a*_1_, …, *a*_*i*_, …, *a*_2^*m*^_}, *i* = 1, 2, …, 2^*m*^.

**Definition 3 (Average Distance).** Given the mobile user *u* and the selection strategy *a* = (*u*_1_, …, *u*_*i*_, …, *u*_*k*_), *i* = 1, 2, …, *k*;*k* ≤ *m*, the average distance between *u* and the cooperative users in *a* is defined as $\bar{dis}(u,a)$.

**Definition 4 (Coverage).** Given the mobile user *u*, the request-response area of *u* is defined as *cover*(*u*).

**Definition 5 (Convex Hull Area).** Given the set of users *users* = {*u*_1_, …, *u*_*i*_, …, *u*_*k*_}, *i* = 1, 2, …, *k*;*k* ≤ *m*, the convex hull area of the user set is defined as *cha*(*u*_1_, …, *u*_*i*_, …, *u*_*k*_).

The actual positioning accuracy generated by a selection strategy *a* is measured by the length of the corresponding actual distance, defined as follows:

**Definition 6 (Actual Distance).** Given the selection strategy *a* = (*u*_1_, …, *u*_*i*_, …, *u*_*k*_), *i* = 1, 2, …, *k*;*k* ≤ *m*, the actual distance is the average distance between the cooperative users and the mobile user *u*, denoted as *ad*(*a*), defined as follows:

$$\begin{array}{c} ad(a)=\bar{dis}(u,a) \end{array}$$ (1)

The inferred positioning accuracy generated by a selection strategy *a* is measured by the size of the corresponding inferred area, defined as follows:

**Definition 7 (Inferred Area).** Given the selection strategy *a* = (*u*_1_, …, *u*_*i*_, …, *u*_*k*_), *i* = 1, 2, …, *k*;*k* ≤ *m*, the inferred area is the intersection of the coverage of the cooperative users, denoted as *ia*(*a*), defined as follows:

$$\begin{array}{c} ia(a)=\overset{k}{\underset{i=1}{⋂}}cover(u_{i}) \end{array}$$ (2)

The privacy generated by a selection strategy *a* is measured by the size of the corresponding privacy area, defined as follows:

**Definition 8 (Privacy Area).** Given the selection strategy *a* = (*u*_1_, …, *u*_*i*_, …, *u*_*k*_), *i* = 1, 2, …, *k*;*k* ≤ *m*, the privacy area is the convex hull area of the cooperative users, denoted as *pa*(*a*), defined as follows:

$$\begin{array}{c} pa(a)=cha(u_{1},\ldots ,u_{i},\ldots ,u_{k}) \end{array}$$ (3)

### Problem hardness

In order to establish the NP-hardness of the BLPPA problem, it is necessary to reduce this problem to an NPC problem. Given that the 3-SAT problem is a known NPC problem, demonstrating that the BLPPA problem can be reduced to the 3-SAT problem will suffice to prove that the BLPPA problem is NP-hard.

**Theorem The BLPPA problem is NP-hard**

**Proof** First, it is necessary to formalize the problem in this paper, with three parameters: *ad*, *pa*, and *ia*, corresponding to actual distance, privacy area, and inferred area in this paper. If ↑ means increase, ↓ means decrease, the simplified constraint relationships among them are as follows:

*ad* ↑ ⇒*pa* ↑, *ia* ↓, meaning an increase in *ad* will lead to an increase in *pa* and a decrease in *ia*.

*pa* ↑ ⇒*ad* ↑, *ia* ↓, meaning an increase in *pa* will lead to an increase in *ad* and a decrease in *ia*.

*ia* ↑ ⇒*pa* ↓, *ad* ↓, meaning an increase in *ia* will lead to a decrease in *pa* and an increase in *ad*.

The ultimate goal is to find values for *ad*, *pa*, and *ia* that satisfy these constraint conditions, with the aim of making *ad* as small as possible to improve actual positioning accuracy, maximizing *ia* to reduce inferred positioning accuracy, and maximizing *pa* to enhance privacy.

For this purpose, it is proposed to create a 3-SAT problem that is equivalent to the problem in this paper. Given that an increase is true and a decrease is false, for each variable *ad*, *pa*, *ia*, introduce corresponding Boolean variables *ad*_*tf*, *pa*_*tf*, *ia*_*tf*. The constructed Boolean expression reflecting the constraint conditions is as follows:

$$\begin{array}{c} \left(ad\_tf\vee pa\_tf\vee \bar{ia\_tf})\wedge (ad\_tf\vee pa\_tf\vee \bar{ia\_tf})\wedge (\bar{ad\_tf}\vee \bar{pa\_tf}\vee ia\_tf\right) \end{array}$$

The Boolean variables that satisfy the objectives are *ad*_*tf* = *false*, *pa*_*tf* = *true*, and *ia*_*tf* = *true*. Substituting these Boolean variables into the aforementioned Boolean expression, the process is as follows:

$$\begin{array}{c} \begin{array}{cc} & \left(ad\_tf\vee pa\_tf\vee \bar{ia\_tf})\wedge (ad\_tf\vee pa\_tf\vee \bar{ia\_tf})\wedge (\bar{ad\_tf}\vee \bar{pa\_tf}\vee ia\_tf\right)\\ & \Rightarrow (flase\vee true\vee true)\wedge (false\vee true\vee \bar{true})\wedge (\bar{false}\vee \bar{true}\vee true)\\ & \Rightarrow true\wedge true\wedge true\\ & \Rightarrow true \end{array} \end{array}$$

The final result shows that the result of Boolean expression is true.

Therefore, it has been proved that the original problem can be reduced to a 3-SAT problem, and the original problem is NP-hard.

## Privacy and positioning accuracy assessment

This section evaluates privacy protection and positioning accuracy respectively.

### Privacy assessment model

**Fig 3:**
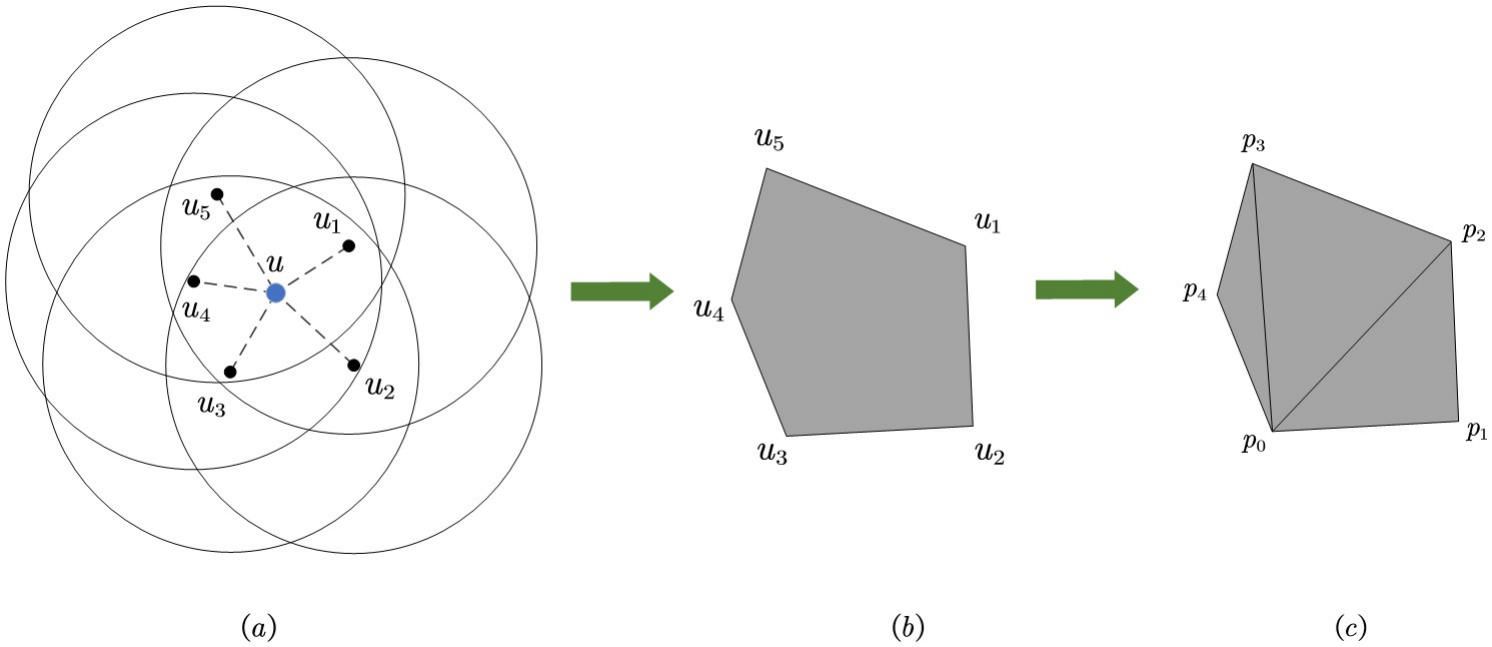
Calculate the privacy area.

Privacy can be measured by the size of the corresponding privacy area. We propose to use the convex hull area composed of cooperative users to quantify the privacy area. In Fig 3(a), mobile user *u* has five cooperating users, namely *u*_1_, *u*_2_, *u*_3_, *u*_4_, and *u*_5_, so a selection strategy of *u* is represented as *a* = (*u*_1_, *u*_2_, *u*_3_, *u*_4_, *u*_5_). Observing Fig 3(a), the privacy area consists of the coordinate points of five cooperative users. The privacy area is extracted from Fig 3(a) to form the convex hull shown in Fig 3(b). Next, the area of this convex hull will be computed.

From Fig 3(b), it can be observed that the convex hull is an irregular polygon. By further subdividing the convex hull, it can be split into several triangles, as shown in Fig 3(c). The area of the convex hull can be calculated by summing the areas of the triangles that compose it. First, determine the set of points *P* = {*p*_2_, *p*_1_, *p*_0_, *p*_4_, *p*_3_} that make up the convex hull. The area of the convex hull formed by this point set is denoted as *cha*(*P*). Select the lower-left point within the convex hull as the reference point, for example, use point *p*_0_ in Fig 3(c) as the reference point. The remaining points are sorted based on the angles formed with the reference point and the positive x-axis. The sorted point coordinates might be represented as {*p*_1_, *p*_2_, *p*_3_, *p*_4_}. The sorted points and reference points form three triangles, and the sum of the areas of the three triangles is exactly equal to the convex hull area, i.e. *conv*(*P*) = *S*(Δ*p*_0_*p*_1_*p*_2_) + *S*(Δ*p*_0_*p*_2_*p*_3_) + *S*(Δ*p*_0_*p*_3_*p*_4_). Below, we will take *S*(Δ*p*_0_*p*_1_*p*_2_) as an example to discuss how to calculate the area of a triangle.

According to the Haversine Formula, the distance between *p*_0_ and *p*_1_ can be calculated based on their longitude and latitude (*lat*_0_, *lng*_0_), (*lat*_1_, *lng*_1_), and (*lat*_2_, *lng*_2_), simply represented as (*x*_0_, *y*_0_), (*x*_1_, *y*_1_), and (*x*_2_, *y*_2_):

$$\begin{array}{c} d(p_{0},p_{1})=2R\cdot \text{arcsin}(\sqrt{{\text{sin}}^{2}\frac{y_{1}-y_{0}}{2}+\text{cos}(y_{0})\cdot \text{cos}(y_{1})\cdot {\text{sin}}^{2}\frac{x_{1}-x_{0}}{2}}) \end{array}$$ (4)

where *R* is the radius of the earth. In the same way, *d*(*p*_0_, *p*_2_) and *d*(*p*_1_, *p*_2_) can be calculated. According to Heron’s Formula, *S*(Δ*p*_0_*p*_1_*p*_2_) can be calculated as:

$$\begin{array}{c} \begin{array}{cc} S(\Delta p_{0}p_{1}p_{2}) & =\sqrt{s(s-d(p_{0},p_{1}))(s-d(p_{0},p_{2}))(s-d(p_{1},p_{2}))}\\ s & =\frac{d(p_{0},p_{1})+d(p_{0},p_{2})+d(p_{1},p_{2})}{2} \end{array} \end{array}$$ (5)

Now, the formula for calculating the privacy area of mobile user *u*’s selection strategy *a* is given:

$$\begin{array}{c} pa(a)=S(\Delta p_{0}p_{1}p_{2})+S(\Delta p_{0}p_{2}p_{3})+S(\Delta p_{0}p_{3}p_{4}) \end{array}$$ (6)

### Positioning accuracy assessment model

There are two types of positioning accuracy: actual positioning accuracy and inferred positioning accuracy. Firstly, the evaluation methods for actual positioning accuracy will be introduced, followed by the evaluation methods for inferred positioning accuracy.

#### Actual positioning accuracy

As introduced in Problem formlation section of this paper, the actual positioning accuracy is measured by the length of the corresponding actual distance, which is the geographic distance between the cooperative user *u*_*i*_ and the mobile user *u*.

**Fig 4:**
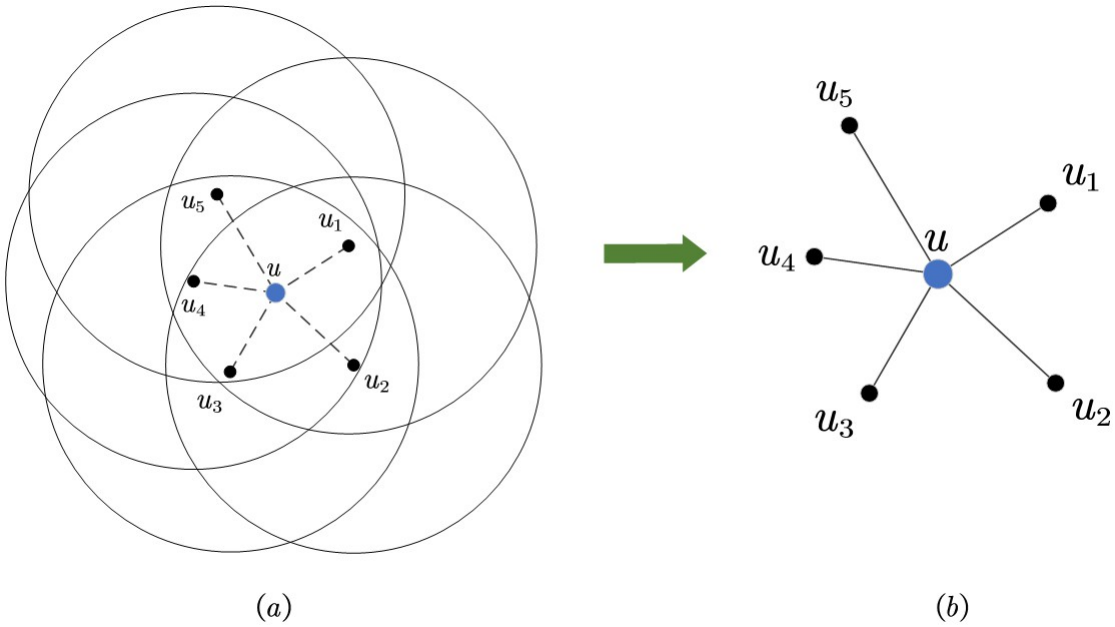
Calculating the actual distance.

In Fig 4(a), mobile user *u* has five cooperative users, namely *u*_1_, *u*_2_, *u*_3_, *u*_4_, and *u*_5_. A selection strategy for *u* is represented as *a* = (*u*_1_, *u*_2_, *u*_3_, *u*_4_, *u*_5_). The dashed line in Fig 4(a) indicates that the cooperative user has a cooperative relationship with the mobile user. Fig 4(b) is a simplified diagram in Fig 4(a), where all cooperative users and mobile users have quantified their cooperative relationships as actual distances, represented by solid lines. According to the Haversine Formula, the actual distance between mobile user *u* and cooperative user *u*_1_ can be calculated based on their latitude and longitude, which are simply denoted as (*x*_*u*_, *y*_*u*_) and $\left(x_{u_{1}},y_{u_{1}}\right)$, and the calculation result is:

$$\begin{array}{c} d(u,u_{1})=2R\cdot \text{arcsin}(\sqrt{{\text{sin}}^{2}\frac{y_{u_{1}}-y_{u}}{2}+\text{cos}(y_{u})\cdot \text{cos}(y_{u_{1}})\cdot {\text{sin}}^{2}\frac{x_{u_{1}}-x_{u}}{2}}) \end{array}$$ (7)

The actual distance calculation method between other cooperative users and the mobile user is similar. Finally, the formula for calculating the average actual distance of the mobile user *u*’s selection strategy *a* is given:

$$\begin{array}{c} ad(a)=\frac{\sum_{i=1}^{k}d(u,u_{i})}{k} \end{array}$$ (8)

#### Inferred positioning accuracy

**Fig 5:**
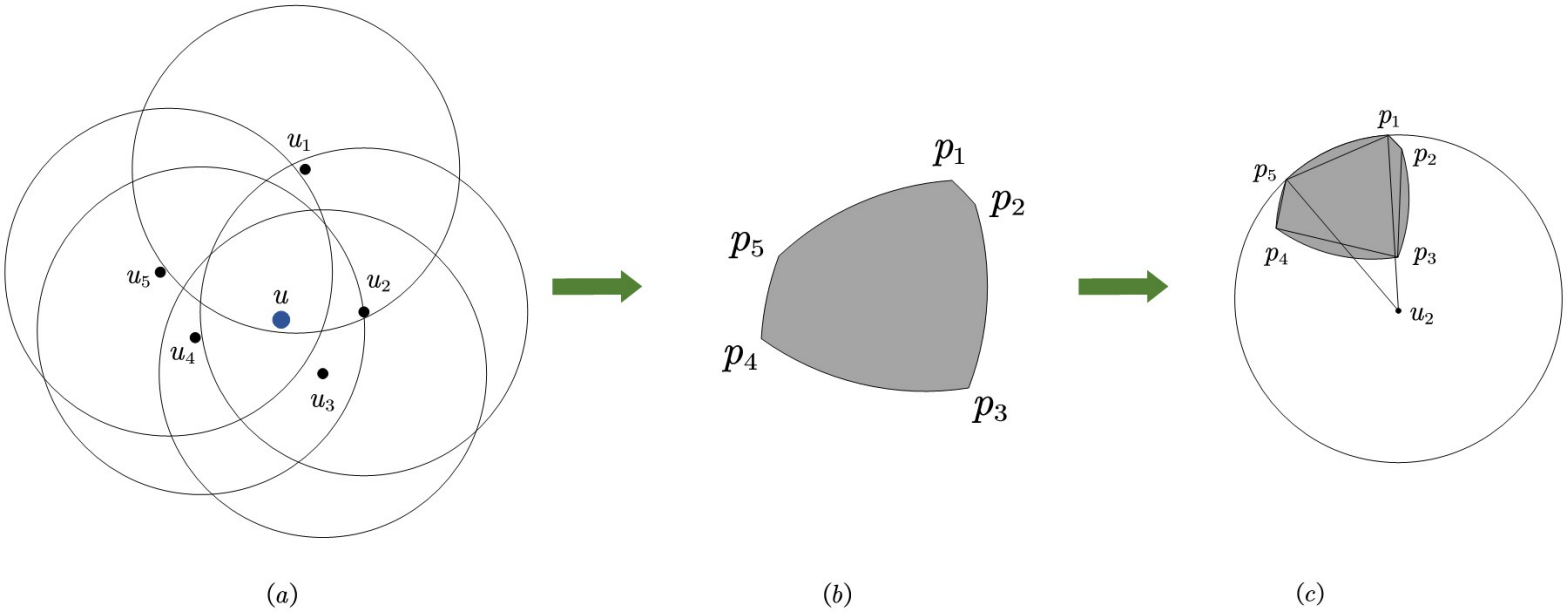
Calculating the inferred area.

The accuracy of inferred positioning is measured by the size of the corresponding inferred area, which is the intersection of the cooperative user coverage. In Fig 5(a), the mobile user *u* has five cooperative users, namely *u*_1_, *u*_2_, *u*_3_, *u*_4_, and *u*_5_. A same selection strategy for *u* is represented as *a* = (*u*_1_, *u*_2_, *u*_3_, *u*_4_, *u*_5_). Extract the inferred area from Fig 5(a) to form the curved polygon shown in Fig 5(b). To calculate the area of the inferred area, the vertex of the curved polygon should be determined first. The method is as follows: calculate the intersection point of each cooperative user and other cooperative users in the requested area, determine whether each intersection point is an inner intersection point. If the distance between the intersection point and each cooperative user is not greater than the radius of the requested area, then the intersection point is considered an inner intersection point, which is a part of the curved polygon point set. Next, we will calculate the area of the curved polygon.

By observing Fig 5(b), it was found that the curved polygon can be divided into a convex hull and multiple arches, as shown in Fig 5(c). Therefore, the area of a curved polygon can be calculated by adding the areas of multiple arches of the convex hull at the center. The method for calculating the area of the convex hull has been detailed in Privacy Assessment Model section, so this section will discuss how to calculate the area of the arch.

Taking the calculation of the arch area $\overset{⌢}{S}(p_{1}p_{5})$ as an example. Firstly, calculating the center angle formed by *p*_1_, *p*_5_, and *u*_2_. If x represents longitude and y represents latitude, then the longitude and latitude of *p*_1_, *p*_5_, and *u*_2_ are simply represented as (*x*_1_, *y*_1_), (*x*_5_, *y*_5_), and $\left(x_{u_{2}},y_{u_{2}}\right)$ respectively. The radius of the cooperative user coverage is *r*. Calculate the center angle *θ*(*p*_1_*u*_2_*p*_5_) as:

$$\begin{array}{c} \theta (p_{1}u_{2}p_{5})=2\text{arcsin}\frac{d(p_{1},p_{5})}{2r} \end{array}$$ (9)

Next, calculate the sector area $\overset{⌢}{S}(p_{1}u_{2}p_{5})$ enclosed by *p*_1_, *p*_5_, and *u*_2_:

$$\begin{array}{c} \overset{⌢}{S}(p_{1}u_{2}p_{5})=\pi r^{2}\frac{\theta (p_{1}u_{2}p_{5})}{360} \end{array}$$ (10)

The triangle area formed by *p*_1_, *p*_5_, and *u*_2_ is $\overset{⌢}{S}(p_{1}u_{2}p_{5})$, and the arch area $\overset{⌢}{S}(p_{1}p_{5})$ can be calculated as:

$$\begin{array}{c} \overset{⌢}{S}(p_{1}p_{5})=\overset{⌢}{S}(p_{1}u_{2}p_{5})-S(\Delta p_{1}u_{2}p_{5})=r^{2}\cdot \text{arcsin}\frac{d(p_{1},p_{5})}{2r}-S(\Delta p_{1}u_{2}p_{5}) \end{array}$$ (11)

Finally, the privacy area calculation formula for *u*’s selection strategy *a* is given:

$$\begin{array}{c} ia(a)=pa(a)+\overset{⌢}{S}(p_{1}p_{2})+\overset{⌢}{S}(p_{2}p_{3})+\overset{⌢}{S}(p_{3}p_{4})+\overset{⌢}{S}(p_{4}p_{5}) \end{array}$$ (12)

### Algorithm analysis

It can be seen from Algorithm 1 that the time complexity of calculating the privacy area is *O*(*n*). And *m* cooperative users can generate *m* − 2 triangles. During the calculation process, the areas of *m* − 2 triangles need to be calculated one by one, and the time required is *O*(*n*).

**Algorithm 1** Privacy Area Calculation

**Input:** The cooperative users *N*(*u*) = {*u*_1_(*x*_1_, *y*_1_), *u*_2_(*x*_2_, *y*_2_), …*u*_*n*_(*x*_*n*_, *y*_*n*_)} for *u*, the selection strategy a, *R*

**Output:** Privacy area *pa*(*a*)

1: K ← 0, *list*_−_*arctan* ← ⊘, *pa*(*a*) ← 0;

2: **for** *u*_*i*_ ∈ {*u*_1_, …, *u*_*n*_} **do**

3:  **if** *y*_*i*_ < *y*_*k*_ or (*y*_*i*_ = = *y*_*k*_ *and x*_*i*_<*x*_*k*_) **then**

4:   *k* = *i*;

5:  **end if**

6: **end for**

7: let *u*_*k*_ be the reference point;

8: swap the positions of *u*_1_ and *u*_*k*_ in *N*(*u*);

9: **for** *u*_*i*_ ∈ {*u*_2_, *u*_3_, …, *u*_*n*_} **do**

10:  calculate the tan value between *u*_1_ and *u*_*i*_: tan(*u*_1_, *u*_*i*_) = (*y*_*i*_ − *y*_1_)/(*x*_*i*_ − *x*_1_);

11:  calculate the arctan value between *u*_1_ and *u*_*i*_: arctan(*u*_1_, *u*_*i*_);

12:  *list*_−_*arctan* ← *arctan*(*u*_1_, *u*_*i*_);

13:  sort the *list*_−_*arctan* by the value of arctan value from smallest to largest;

14: **end for**

15: **for** *p*_*i*_ ∈ {*u*_2_, *u*_3_, …, *u*_*n*_} **do**

16:  calculate the distances *d*(*u*_1_, *u*_*i*_) between *u*_1_ and *u*_*i*_, *d*(*u*_1_, *u*_*i*+1_) between *u*_1_ and *u*_*i*+1_, and *d*(*u*_*i*_, *u*_*i*+1_) between *u*_*i*_ and *u*_*i*+1_ according to formula (8);

17:  calculate *S*(△*u*_1_*u*_*i*_*u*_*i*+1_) according to formula (6);

18:  *pa* ← *pa* + *S*(△*u*_1_*u*_*i*_*u*_*i*+1_);

19: **end for**

20: return *pa*(*a*);

From Algorithm 2, it can be seen that the time complexity of calculating the actual distance is also *O*(*n*). In the calculation process, the geographic distances of *m* cooperative users and requesting users need to be calculated separately, and then the mean value is calculated, which requires a time overhead of *O*(*n*).

**Algorithm 2** Actual Distance Calculation

**Input:** The cooperative users *N*(*u*) = {*u*_1_(*x*_1_, *y*_1_), *u*_2_(*x*_2_, *y*_2_), …*u*_*n*_(*x*_*n*_, *y*_*n*_)} for mobile user *u*(*x*, *y*), the selection strategy *a*, *R*

**Output:** Actual Distance *ad*(*a*)

1: *ad*(*a*)←0, *dis* ← 0;

2: **for** *u*_*i*_ ∈ {*u*_1_, *u*_2_, …, *u*_*n*_} **do**

3:  calculate the distances *d*(*u*, *u*_*i*_) between *u* and *u*_*i*_ according to formula (8);

4:  *dis* ← *dis* + *d*(*u*, *u*_*i*_);

5: **end for**

6: calculate average distance *ad*(*a*): $ad(a)=\bar{dis}(u,\{u_{1},u_{2},\ldots ,u_{k}\})=\frac{dis}{n}$;

7: return *ad*(*a*);

It can be seen from Algorithm 3 that the time complexity of calculating the size of the inferred area is *O*(*kn*^2^). We assume that the coverage of all users is a circle. The analysis steps are as follows:

Step 1: The coverage of any two cooperative users will produce two intersection points. The time complexity required to calculate all intersection points of the coverage of *m* cooperative users is *O*(*n*^2^).

Step 2: If the distance between the intersection point and all cooperative users is not greater than the radius, the intersection point is regarded as an internal intersection point, and the time complexity required to determine whether all intersection points are internal intersection points is *O*(*n*^2^).

Step 3: Calculate the convex hull area formed by all internal intersection points according to Algorithm 1, the required time complexity is *O*(*n*).

Step 4: Two adjacent inner intersection points form an arch, and the time complexity required to calculate the areas of all arches is *O*(*n*).

**Algorithm 3** Inferred Area Calculation

**Input:** The cooperative users *N*(*u*) = {*p*_0_(*x*_0_, *y*_0_), *p*_1_(*x*_1_, *y*_1_), …*p*_*n*_(*x*_*n*_, *y*_*n*_)} for *u*, the coverage radius *r* of the cooperative user, the selection strategy *a*, *R*

**Output:** Inferred Area *sa*(*a*)

1: determine all circles {*circes*(*u*), *circes*(*u*_1_), …, *circes*(*u*_*n*_)};

2: *candidate* ← ⊘, *result* ← ⊘, $\overset{⌢}{S}(result)\leftarrow 0$, *sa*(*a*) ← 0;

3: **for** *circles*(*u*_*i*_) ∈ {*circles*(*u*_1_), *circles*(*u*_2_), …, *circles*(*u*_*n*_)} **do**

4:  **for** *circles*(*u*_*j*_) ∈ {*circles*(*u*_*i*+1_), …, *circles*(*u*_*n*_)} **do**

5:   calculate intersection points of *circles*(*u*_*i*_) and *circles*(*u*_*j*_): *p*_0_,*p*_1_;

6:   *candidate* ← {*p*_0_, *p*_1_};

7:  **end for**

8: **end for**

9: **for** *p*_*i*_ ∈ *candidate* **do**

10:  **for** *circles*(*u*_*k*_) ∈ {*circles*(*u*_1_), *circles*(*u*_2_), …, *circles*(*u*_*n*_)} **do**

11:   calculate the distance between *p* and *circles*(*u*_*k*_):*d*(*p*_*i*_, *circles*(*u*_*k*_));

12:   **if** *d*(*p*_*i*_, *circles*(*u*_*k*_)) ≤ *r* **then**

13:    *result* ← *p*_*i*_;

14:   **end if**

15:  **end for**

16: **end for**

17: calculate the convex hull area *area*(*result*) of *result* by **Algorithm 1**;

18: **for** *p*_*i*_ ∈ *result* **do**

19:  calculate the central angle of *p*_*i*_, *p*_*i*+1_, $O_{p_{i}p_{i+1}}$: $\theta (p_{i}O_{p_{i}p_{i+1}}p_{i+1})\leftarrow 2arcsin(d(p_{i},p_{i+1})/2r)$;

20:  calculate the sector area of *p*_*i*_, *p*_*i*+1_, $O_{p_{i}p_{i+1}}$: $\overset{⌢}{S}(p_{i}O_{p_{i}p_{i+1}}p_{i+1})\leftarrow \pi r^{2}(\theta (p_{i}O_{p_{i}p_{i+1}}p_{i+1})/360)$;

21:  calculate *S*(△*p*_0_*p*_*i*_*p*_*i*+1_) according to formula (6);

22:  calculate the arch area: $\overset{⌢}{S}(p_{i}p_{i+1})\leftarrow \overset{⌢}{S}(p_{i}O_{p_{i}p_{i+1}}p_{i+1})-S(⌢p_{i}O_{p_{i}p_{i+1}}p_{i+1})$;

23:  $\overset{⌢}{S}(result)\leftarrow \overset{⌢}{S}(result)+\overset{⌢}{S}(p_{i}p_{i+1})$;

24: **end for**

25: $sa\left(a\right)\leftarrow \overset{⌢}{S}(result)+area(result)$;

26: return *sa*(*a*);

### Optimization function

To achieve a better balance among privacy area, actual positioning accuracy, and inferred positioning accuracy, it is necessary to select collaborative users in the optimal selection strategy to construct an anonymous area. The formation of optimal selection strategy must satisfy the following conditions:

In order to maximize the actual positioning accuracy, it is necessary to minimize the average distance *ad*(*a*) between each collaborative user and the requesting user. Given *k* collaborative users for mobile user *u*, the optimization function can be described as:

$$\begin{array}{c} \begin{array}{cc} ad(a) & =\underset{a\in a}{\text{min}}\;\;ad(a)\\ & =\underset{a\in a}{\text{min}}\frac{\sum_{i=1}^{k}d(u,u_{i})}{k} \end{array} \end{array}$$ (13)

where the BLPPA strategy, denoted as **a**, consists of several selection strategies *a*.

To maximize the reduction of inferred positioning accuracy, it is necessary to maximize the common coverage area *ia*(*a*) among the collaborative users. Given *k* collaborative users for mobile user *u*, denoted as {*u*_1_, *u*_2_, …, *u*_*k*_}, the optimization function can be described as:

$$\begin{array}{c} \begin{array}{cc} ia(a) & =\underset{a\in a}{\text{max}}\;\;ia(a)\\ & =\underset{a\in a}{\text{max}}(\sum_{i=1}^{k-2}S(\Delta u_{0}u_{i}u_{i+1})+\sum_{i}^{k-1}\overset{⌢}{S}(u_{i}u_{i+1})) \end{array} \end{array}$$ (14)

To maximize the privacy area, it is necessary to maximize the convex hull area *pa*(*a*) formed by the collaborative users. Similarly, given *k* collaborative users for mobile user *u*, the optimization function can be described as:

$$\begin{array}{c} \begin{array}{cc} pa(a) & =\underset{a\in a}{\text{max}}\;\;pa(a)\\ & =\underset{a\in a}{\text{max}}\;\;\sum_{i=1}^{k-2}S(\Delta u_{0}u_{i}u_{i+1}) \end{array} \end{array}$$ (15)

If the actual distance is minimized, the inferred area is maximized, and the privacy area is maximized at the same time, the optimal strategy selection problem is transformed into a multi-objective optimization problem.

$$\begin{array}{c} \underset{a}{\text{min}}\{\lambda_{ad}\cdot \underset{a\in a}{ad}(a)+\lambda_{ia}\cdot \underset{a\in a}{ia}(a)+\lambda_{pa}\cdot \underset{a\in a}{pa}(a)\} \end{array}$$ (16)

To address the above multi-objective optimization problem, we transform the multi-objective optimization problem into a single objective optimization problem by assigning weights to each objective function according to the relationship between the objective functions. Specifically, the multiple objective functions are linearly combined into a Privacy Positioning Accuracy Weighted Average (PPAWA) optimization function, and the optimal selection strategy is sought by adjusting the weights of each objective. Combining Eqs (13)–(16), the privacy positioning accuracy weighted average optimization function can be described as:

$$\begin{array}{c} ppawa(a)=1-\underset{a}{\text{min}}\{\lambda_{ad}\cdot \underset{a\in a}{ad}(a)+\lambda_{ia}\cdot \underset{a\in a}{ia}(a)+\lambda_{pa}\cdot \underset{a\in a}{pa}(a)\} \end{array}$$ (17)

where λ_*ad*_, λ_*ia*_, and λ_*pa*_ represent the weights assigned to the actual distance, inferred area, and privacy area, respectively, indicating their relative importance in the BLPPA problem.

This method of solving the optimal strategy for the BLPPA problem is hereinafter referred to as Balance Location Privacy and Positioning Accuracy-Actual distance and Inferred area and Privacy area(BLPPA-AIP).

## Experimental analysis

We conducted a large number of experiments to test the performance of BLPPA-AIP, and selected five methods for comparison, as shown in Table 3.

**Table 3:** Methods and descriptions of comparisons.

Methods	Description
BLPPA-AIP	Our method attempts to find the optimal selection strategy
APA(Actual Positioning Accuracy)	Only considering improving actual positioning accuracy
IPA(Inferred Positioning Accuracy)	Only considering reducing the accuracy of inferred positioning
PA(Privacy Area)	Only consider maximizing privacy area
RANDOM	Randomly select *k* cooperative users from *n* to cooperate
GREEDY	Select all cooperative users to cooperate with

### Experimental setup and performance metrics

The performance of BLPPA-AIP is tested using the publicly available dataset from the Geolife project of Microsoft Research Asia. In order to simulate different scenarios, we set up three scenarios: sparse scenario, normal scenario, and dense scenario, and the number of users in different scenarios is set to 100, 200, and 300, respectively [34], with the number of cooperative users taking the value of 10, and the radius of the user’s coverage is 250 meters. In each experiment, given the location of a mobile user, we use a different approach to formulate a selection strategy for the mobile user. The weights of privacy, actual location accuracy, and inferred location accuracy are all set to 1/3, giving all three equal importance.

For effectiveness assessment, four metrics are used: PPAWA, actual positioning accuracy, inferred positioning accuracy, and privacy area. To assess the efficiency, experimental comparisons are made with other methods, which are analyzed in terms of computation time and delay distance, respectively.

### Experimental results

#### Effectiveness

Tables 4–6 compare the average of privacy positioning accuracy, actual positioning accuracy, inferred positioning accuracy, and privacy area of six methods (i.e., BLPPA-AIP, APA, IPA, PA, EANDOM, GREEDY). Overall, compared to the other five methods, BLPPA-AIP performs best: it has the highest average PPAWA, the second largest inferred area, the second shortest average distance, and the fourth largest privacy area. This indicates that the scheme achieves an appropriate balance between location privacy and positioning accuracy.

**Table 4:** Dense scenarios.

Method	PPAWA	Actual Distance	Inferred Area	Privacy Area
BLPPA-AIP	**0.7138**	81.8627	64457.1538	11418.17
APA	0.5259	**74.5726**	35434.4131	2369.17
IPA	0.544	121.0637	**68397.4418**	635.31
PA	0.4981	162.5725	2425.916	**39633.1**
RANDOM	0.5233	139.5602	2425.916	**39633.1**
GREEDY	0.524	153.4682	50463.9713	13804.49

**Table 5:** Ordinary scenarios.

Method	PPAWA	Actual Distance	Inferred Area	Privacy Area
BLPPA-AIP	**0.7542**	118.519	47555.8488	30688.73
APA	0.5632	**109.1796**	33844.5029	10564.84
IPA	0.5697	176.1167	**76717.4008**	3791.02
PA	0.6023	155.5322	8047.4802	**42536.65**
RANDOM	0.615	147.5397	8047.4802	**42536.65**
GREEDY	0.5314	156.8365	5190.5412	35328.99

**Table 6:** Sparse scenarios.

Method	PPAWA	Actual Distance	Inferred Area	Privacy Area
BLPPA-AIP	**0.707**	183.9408	31842.6171	19198.71
APA	0.5873	**174.4127**	21696.7215	9048.57
IPA	0.6395	203.0802	**31981.3572**	6456.25
PA	0.5966	226.5934	643.3908	**108453.87**
RANDOM	0.6107	214.7921	643.3908	**108453.87**
GREEDY	0.5894	224.0338	685.1998	105014.16

From the PPAWA column in Tables 4–6, it can be seen that BLPPA-AIP achieved the highest PPAWA values in all three scenarios. In Table 4, the average PPAWA values are 26.32%, 23.79%, 30.22%, 26.69%, and 26.59% higher than APA, IPA, PA, RANDOM, and GREDDY, respectively. This indicates that BLPPA-AIP can best balance location privacy and positioning accuracy. Meanwhile, from the Actual Distance column in Tables 4–6, it can be seen that the average distance generated by BLPPA-AIP to measure actual positioning accuracy is the second shortest among all methods, only 9.78% higher than the APA. From the Inferred Area column, it can be seen that the inferred area generated by BLPPA-AIP for measuring the accuracy of inferred positioning is the second largest among all methods, only 5.76% lower than the IPA. From the Privacy Area column, it can be seen that the comparison of privacy area sizes shows that BLPPA-AIP is worse than most methods and 71.19% lower than the PA. This result is expected, as APA, IPA, and PA only consider actual positioning accuracy, reduced inferred positioning accuracy, and privacy areas, respectively. When it is necessary to balance actual positioning accuracy, reduce inferred positioning accuracy, and privacy areas, these three methods are not suitable.

Take the performance of PA in ordinary scenarios as an example. Although PA achieves the maximum privacy area (Table 5), this method achieves the smallest inferred area and the longest average distance. This indicates that maximizing the user’s privacy area without considering actual and inferred positioning accuracy will put mobile users at risk of location privacy leakage and reduced positioning accuracy. Similarly, the balance between position privacy and positioning accuracy achieved by APA is also extremely uneven. As shown in Table 5, although APA achieved the shortest average distance of 102.39 m, its privacy area is the second smallest, with 10564.84 m^2^. The reason is that the goal of APA is to maximize the actual positioning accuracy, and in this process, it is inevitable to overlook the consideration of inferred positioning accuracy and privacy areas. Similar to APA, although IPA achieved the maximum inferred area of 76717.40 m^2^, it had the longest average distance and the smallest privacy area, with 176.1167 m and 3791.02 m^2^, respectively. The reason is that the goal of IPA is to minimize the actual inferred positioning accuracy, without considering the actual positioning accuracy and privacy area. This indicates that although APA and IPA can ensure the positioning accuracy of mobile users, they can easily lead to the leakage of user location privacy.

From Tables 4–6, it can be seen that all six methods have achieved smaller average distances, smaller inference areas, and smaller privacy areas in dense scenarios. This indicates that in dense scenarios, protecting the location privacy of mobile users is relatively difficult. However, dense scenes have the most mobile users and environmental information, and mobile users can infer corresponding locations and points of interest based on this. Therefore, in such dense scenarios, BLPPA-AIP is the best method because it provides a very high level of positioning accuracy without sacrificing too much location privacy.

#### Efficiency

*1) Computing time*. Due to the NP-hardness of the BLPPA problem, given mobile users in a specific area, if there are many cooperative users to choose from in the area, the time complexity of finding the selection strategy is high. Assuming there are *m* cooperative users around *u*, then there are a total of 2^*m*^ possible selection strategies. To evaluate the efficiency of BLPPA-AIP, we will discuss the computational time required to find a solution to the BLPPA problem.

Table 7 shows the calculation time evaluation results. The main reason why BLPPA-AIP takes the most time is that it needs to calculate all possible 2^*m*^ selection strategies for *m* cooperative users for a given mobile user *u*, and select the best selection strategy among them. And the result of calculation time 77.795 ms is acceptable.

**Table 7:** Computing time.

Method	BLPPA-AIP	APA	IPA	PA	GREEDY	RANDOM
Time (ms)	77.795	12.988	60.840	19.971	10.995	10.995

APA, IPA and PA take more time than the RANDOM and GREEDY with the goal of minimizing the average distance, maximizing the inferred area and maximizing the privacy area, respectively. The reason is because APA, in pursuit of minimizing the distance, only needs to select a cooperative user closest to *u*. Similarly, to maximize the privacy area, PA can simply select all cooperative users. These two methods do not need to compute all possible selection strategies as BLPPA-AIP does. In contrast, IPA pursues maximizing the inferred area and needs to compute all possible selection strategies like BLPPA-AIP, but it takes less time than BLPPA-AIP because it considers fewer factors. The reason that the GREEDY takes less time is because it selects all cooperative users and performs the computation of only one selection strategy. The reason that the RANDOM takes less time is because the RANDOM takes less time because it randomly selects one selection strategy and also performs the computation of only one selection strategy.

**Fig 6:**
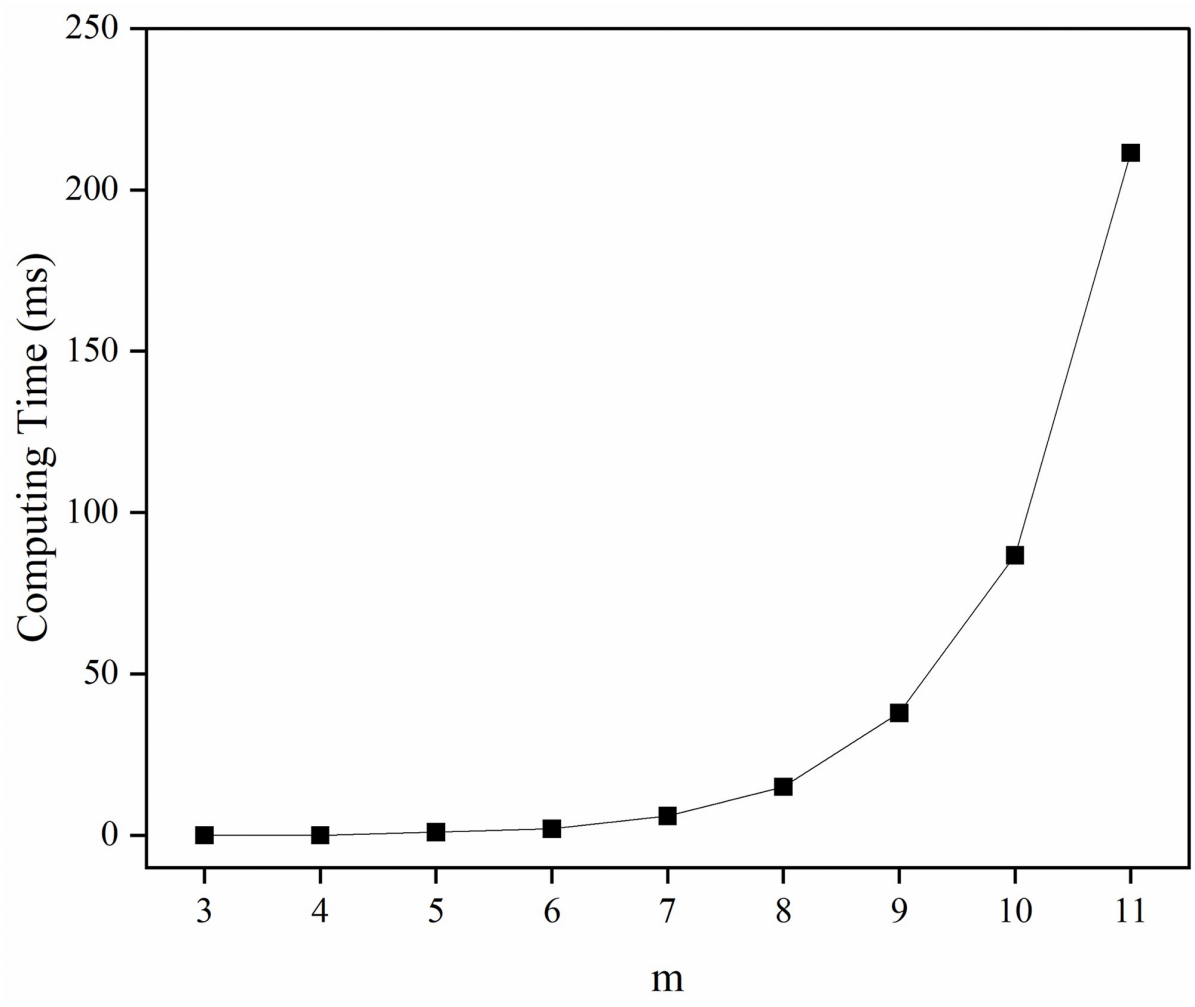
Computing time for different *m*.

Fig 6 illustrates the effect of different numbers of cooperative users on the computation time. It can be seen that the computation time increases exponentially as the value of *m* increases, this is because the method BLPPA-AIP we proposed needs to compute all possible 2^*m*^ selection strategies for *m* cooperative users for cooperation given a mobile user *u* and select the best selection strategy among them. So when the value of *m* increases, the number of selection strategies that may need to be computed grows exponentially, which greatly increases the amount of computation and also leads to an exponential increase in computation time. From Fig 6, it can be seen that when the number of required cooperative users is small (e.g., when *m* is 3 to 7), the computation time is low, but fewer cooperative users produce weaker privacy protection. When the number of required cooperative users is large (e.g., when *m* is greater than 10), the computation time is too high to be acceptable, even though the privacy protection is excellent. Therefore, the value of *m* should be moderate, and all the experiments in this paper are done when *m* is 10.

*2) Delay distance* Delay distance is the combined measure of delay and distance experienced by the user in the moving process, which is specifically described as the user’s moving distance from issuing a cooperation request to balancing location privacy protection and positioning accuracy, which reflects the efficiency of the method used for balancing location privacy protection and positioning accuracy, and the shorter the delay distance is, it indicates that the method handles balancing location privacy protection and positioning accuracy more efficiently in the process of the user’s moving.

We set three speed metrics: 5km/h, 30km/h and 60km/h, which are used to simulate the user’s movement at slow, medium, and fast speeds, respectively [35]. Table 8 demonstrates the comparison results of the delay distance of each method at different speeds. The delay distances of BLPPA-AIP methods are higher than the comparison methods at different speeds, but the delay distances of BLPPA-AIP methods at different speeds are within the acceptable range.

**Table 8:** Comparison of delay distance for each method at different speeds (m).

Method	5km/h	30km/h	60km/h
BLPPA-AIP	0.15657	0.93942	1.87883
APA	0.01943	0.11656	0.23312
IPA	0.11363	0.68178	1.36355
PA	0.03324	0.19945	0.39890
GREEDY	0.01527	0.09164	0.18328
RANDOM	0.01527	0.09164	0.18328

When the user moves at a slow speed (e.g., walking), the delay distance of BLPPA-AIP is 0.15657 m, which is only 0.14 m lower than that of the GREEDY and RANDOM methods, which have the shortest delay distances at the same speed, indicating that the delay distance of the BLPPA-AIP method does not affect the user’s experience of the location-based service under the user’s slow speed movement.

When the user moves at medium speed (e.g., riding), the delay distance of BLPPA-AIP is 0.93942 m, which is 0.85 m lower than that of GREEDY and RANDOM, which have the shortest delay distances at the same speed, indicating that under the user’s medium-speed movement, the delay distance of the BLPPA-AIP method basically does not affect the user’s experience of location-based services.

When the user is moving fast (e.g., driving), the delay distance of BLPPA-AIP is 1.87883 m, which is 1.69 m lower than that of the GREEDY and RANDOM methods that have the shortest delay distances at the same speed, indicating that under the user’s fast movement, the delay distance of the BLPPA-AIP method basically does not affect the user’s experience of the location-based service.

## Conclusion

In this paper, we propose a balanced location privacy and positioning accuracy strategy BLPPA-AIP. In terms of location privacy protection, BLPPA-AIP provides personalized location privacy protection for mobile users by constructing an anonymous area in the edge environment. In terms of improving location accuracy, BLPPA-AIP models the problem of selecting cooperative users as an objective optimization problem and constructs an optimization function to select cooperative users to ensure the highest location accuracy. In addition, we also propose methods to evaluate location privacy, actual positioning accuracy, and inferred positioning accuracy, so that BLPPA-AIP can achieve a balance between location privacy and positioning accuracy. Simulation results show that BLPPA-AIP not only achieves better location privacy, but also ensures high positioning accuracy, i.e., it strikes a proper balance between location privacy and positioning accuracy. In future work, how to further improve the efficiency as well as analyze the evaluation metrics affecting the choice of cooperative users at a finer granularity will be the next research focus.
